# Serum AMH level as a marker of acute and long-term effects of chemotherapy on the ovarian follicular content: a systematic review

**DOI:** 10.1186/1477-7827-12-26

**Published:** 2014-03-26

**Authors:** Maëliss Peigné, Christine Decanter

**Affiliations:** 1 Department of Endocrine Gynaecology and Reproductive Medicine, Hôpital Jeanne de Flandre, CHRU, Lille 59037, France

**Keywords:** AMH, MIS, Cancer, Chemotherapy, Fertility preservation, Follicles, Ovarian toxicity

## Abstract

Anti-Müllerian hormone (AMH) is a very sensitive indicator of the ovarian follicular content. Chemotherapeutic agents are notoriously ovariotoxic in that they damage follicles. The aim of this systematic review was to investigate the interest of serum AMH variations in determining the acute and long-term effects of chemotherapy on the ovarian reserve. According to the PRISMA guidelines, searches were conducted on PubMed for all English language articles until December 2013. Fifteen articles that focused on dynamic variations of AMH levels before and after chemotherapy were selected. Cancer patients have significantly lower AMH after chemotherapy than age-matched controls. Longitudinal studies of AMH variations before, during and after chemotherapy provide information about the degree of follicle loss for each patient according to different chemotherapy regimens. Different patterns of AMH levels during the ovarian recovery phase make it possible to discriminate between high and low gonadotoxic chemotherapy protocols. In addition, pretreatment AMH levels are shown to predict the long-term ovarian function after the end of treatment. These results may help to better understand the ovarian toxicity mechanisms of chemotherapy and to predict the degree of the ovarian follicle loss. Therefore, it can be useful for fertility preservation strategies, fertility counseling and future family planning.

## Background

With the continuous improvement of cure rate over the last few decades, ovarian function resurgence and reproductive capacity after cancer treatment have become important quality-of-life issues. Chemotherapy regimens, particularly those including alkylating agents, are notoriously ovariotoxic by damaging all kinds of follicles from primary to preantral and antral stages [1-4]. The precise mechanisms of this toxicity are uncertain. Increased apoptotic processes seem to be the main factor [3,5-9], although cortical fibrosis and blood vessel injury are also described [10,11]. It is now well-established that the degree of this ovarian toxicity is highly dependent on age, treatment and dosage [5,12]. Ovarian damage can be permanent, particularly in the case of protocols including alkylating agents, leading to premature ovarian failure and/or infertility [2,3,13]. In other cases, recovery of ovarian function may occur.

### Chemotherapy and ovarian follicles

The toxicity of alkylating agents has been well studied. Experimental studies in rats and rhesus monkeys, treated with cyclophosphamide, have shown that the depletion of primordial follicles is rapid and drastic [14-16]. Human ovarian histology following chemotherapy treatments evidenced atrophy, reduced follicle store [5,10,17] and especially, primordial follicle pool loss [18,19]. Furthermore, one of these studies reported a deep AMH decrease in mice treated with cyclophosphamide concomitantly with the histological follicular depletion [16]. Some authors suggest that this primordial follicle loss can arise in two different ways. The first would be a direct eliminatory effect of chemotherapeutic agents on primordial follicles. The second would be indirect, through an excessive recruitment of the primordial follicles into the growing pool due to a decrease in Anti-Müllerian Hormone (AMH) after growing follicle damage [7]. Indeed, it has been shown that AMH-knockout mice exhibit premature activation and rapid depletion of ovarian follicle reserve [20]. In addition, a recent study showed that cyclophosphamide triggers upregulation of the PI3K pathway, initiating a wave of follicle recruitment and growth, and ultimately, burnout of the ovarian follicle reserve [21].

Oocyte and somatic cells in the ovary can be potential targets of chemotherapeutic agents, with differential sensitivity to the various types of drugs. In animal models, cyclophosphamide is well known to induce apoptosis of pregranulosa and granulosa cells in all classes of follicles [22]. Oocytes are damaged only in primordial and small antral follicles [14,22]. A recent study in mice investigated the effect of doxorubicin on the ovarian follicles and suggested that damage to the oocytes may be mainly due to somatic cell failure [23]. Using a human ovarian xenografting model, Oktem and Oktay [8] showed that primordial follicle density is rapidly affected by apoptosis after cyclophosphamide injection (12%, 53% and 93% of follicle loss at 12, 24 and 48 h after the first injection, respectively). Human oocytes are drastically damaged (100% at 12 h post injection), followed by granulosa cells (63% at 12 h). The fact that alkylating agents are not cell-cycle specific and thus, do not require cell proliferation for their cytotoxic action can explain why resting follicles are also damaged. While the follicular toxicity of alkylating agents such as cyclophosphamide is well defined [2,3,13], that of multi-agent regimens remains poorly documented.

In addition to the apoptotic phenomenon of granulosa cells, ovarian stromal tissue could also be damaged by chemotherapy due to cortical fibrosis and blood vessel injury [7,10,11]. These phenomena could participate secondarily to the follicular alteration through impairment of follicular vascularization.

Most of the studies that have addressed the question of reproductive capacity after cancer have focused on markers such as cycle length, pregnancy occurrence and/or basal FSH values. Nevertheless, recent studies have shown that follicular depletion may occur despite recovery of regular menstrual cycles [24-26]. This suggests that more accurate indicators are needed in order to properly inform the women about their fertility after treatment. Recent studies have suggested that AMH could be a valuable indicator of follicular depletion in breast cancer [27-29] and lymphoma patients [24].

### AMH and follicles

AMH is a dimeric glycoprotein and belongs to the TGF β family which acts on tissue growth and differentiation. Its name comes from its first known function: the regression of Müllerian ducts during male fetal differentiation. In women, serum AMH appears to be exclusively of ovarian origin since AMH is undetectable in serum 3 to 5 days following bilateral ovariectomy [30]. AMH is produced by granulosa cells of preantral and small antral follicles and its main physiological role seems to be the inhibition of the initial follicular recruitment from the primordial to the antral pool [31]. It has been extensively studied in Assisted Reproductive Therapy processes. It is now well established that AMH is the more accurate marker of the ovarian reserve [32]. In women, serum AMH levels are almost undetectable at birth and progressively increase to the adult level. It appears to be stable up to 25–30 years and then to decrease throughout the remaining reproductive life until being undetectable after spontaneous menopause [30,32-36]. AMH level on day 3 of the menstrual cycle has been shown to be strongly correlated with the antral follicle count (AFC) [33] and with the primordial follicule pool [37-41]. Furthermore, AMH measurement is reproducible and easy to obtain. Highly sensitive ELISA assays are currently available to measure AMH levels in women: the Gen II assay and the Immunotech MIS-AMH assay. Both are produced by the same company (Beckman Coulter). They use different monoclonal antibodies targeting different regions of AMH and different calibrators thus explaining that the two assays generate quite different results and are not interchangeable. The Diagnostic Systems Laboratories (DSL) assay is not used anymore, but has been used in several studies cited in this review.

Finally, as AMH concentration does not change significantly during the menstrual cycle [30,42,43] and is weakly influenced by short term (< 6 months) gonadotropin suppressing treatments [44,45] unlike other follicular markers such as FSH, estradiol, AFC, Inhibin B [30,42,43], AMH is thus considered as the more specific and reproducible marker of the ovarian reserve.

This review aims to highlight whether serum AMH could be a valuable indicator of the acute and long-term effects of chemotherapy regimen on the ovarian follicular content and how it could help in better understanding the chemo-induced toxicity and in fertility preservation strategies.

## Methods

A systematic MEDLINE (PubMed) search was performed regarding articles published in English containing key words “AMH” (Anti-Müllerian hormone), “MIS” (Müllerian Inhibiting Substance), “chemotherapy”, “follicle”, “ovarian reserve”, “ovarian toxicity” and “alkylating agents”. All the relevant publications were selected until December 2013. We examined the 36 published studies measuring AMH levels in women who have undergone chemotherapy for different types of cancer. As this systematic review aims to focus on dynamic variations of AMH levels prior to, during and after chemotherapy, we excluded those that investigated only the post-treatment AMH levels compared to age-matched controls. 15 articles were then taken into consideration for this review. All studies are prospective observational studies including pre and post-chemotherapy AMH level measurement analysis.

## Results and discussion

### AMH pre/post chemotherapy

It has been shown that post-chemotherapy AMH levels are significantly lower than those of age-matched controls confirming the idea that AMH is a more accurate, direct and specific indicator of the follicle loss. Nevertheless, only the comparison of pre/post-chemotherapy AMH levels would allow the evaluation of the individual degree of follicular depletion. Studies which addressed this issue are summarized in Table 1.

**Table 1:** Summary of studies with pre/post-treatment AMH measurements in young women undergoing chemotherapy (CT)

**Study**	**Cancer**	**Number of patients**	**Conclusion**
Lutchman-Singh, 2007 [46]	Breast	22	-AMH post-CT < AMH pre-CT
			-AMH pre-CT = AMH in age-matched controls
Lie Fong, 2008 [49]	Haematological	25	-AMH post-CT < AMH pre-CT
			-AMH pre-CT < AMH in older controls
Aslam, 2011 [47]	All types	8	-AMH post-CT < AMH pre-CT
			-AMH post-CT < AMH in age-matched controls with the same follow-up
Lawrenz, 2012 [48]	Lymphoma	38	-AMH post-CT < AMH pre-CT
			-AMH pre-CT < AMH in age-matched controls
			-Number of retrieved oocyte for cryopreservervation in lymphoma < retrieved oocytes in women with breast cancer

In 22 young, regularly menstruating women with breast cancer, Lutchman-Singh et al. [46] demonstrated that, for each woman, the post-chemotherapy serum AMH level was significantly lower than the pre-treatment level. In this study, pre-treatment AMH levels were not different between the patients and the control population. Aslam et al. [47] in a smaller case–control study had the same results. In contrast, two other studies [48,49] concluded that ovarian reserve seemed to be already compromised prior to chemotherapy for haematological malignancy. Indeed, in both these studies AMH was significantly lower in the treated group before chemotherapy compared to controls, even though the latter group was older. In addition, Lawrenz et al. [48] reported that the number of retrieved oocytes for fertility preservation was significantly lower in patients with lymphoma compared to those with breast cancer. They hypothesized that haematological malignancies may affect the ovarian reserve in women before chemotherapy, as it was already suggested in men [50] for semen quality, through unknown mechanisms.

### AMH prior to, during and after chemotherapy

To date, few studies have been designed to prospectively evaluate the longitudinal variations of AMH levels in patients treated by chemotherapy. This approach allows for studying the timing of follicle loss and of putative recovery. Five of these studies have been conducted in pre-menopausal, breast cancer patients [26-29,51], one in lymphoma patients [24], two in several types of cancer [52,53] and two in childhood cancer [54,55]. These studies are summarized in Table 2 for adult cancers and Table 3 for childhood cancers.

**Table 2:** Summary of studies with longitudinal follow-up of serum AMH levels in young women undergoing chemotherapy (CT) for adult cancer

**Study**	**Cancer**	**Treatment**	**Age (range)**	**Times of AMH measurement**	**Number of patients**	**Conclusions about AMH follow up**
**Anderson, 2006 [**[28]**]**	Early stage breast cancer	7 protocols all including alkylating agents	41 (28–52)	Before CT, every 3 months during CT	56	-Significant fall of AMH levels 3 months after the start of CT.
						-Undetectable values of AMH in most patients at the end of CT.
						-Different degrees of gonadotoxicity according to protocols.
**Anders, 2008 **[27]	Early stage breast cancer	Anthracycline, cyclophosphamide and taxane	40 (21–51)	Before CT, 3 to 6 weeks post CT, 6 months and year post CT	38	-Undetectable values of AMH in most patients at the end of CT and one year post-CT.
						-Pre-CT AMH levels lower among women who became amenorrheic compared to those who resumed menses.
**Decanter, 2010 **[24]	Lymphoma	Alkylating or non Alkylating regimens	24 (18–32)	Before CT, 15 days after first cycle, 15 days before last cycle, every 3 months after CT until 1 year	30 (17 non alkylating protocol, 13 alkylating protocol)	No difference between protocols regarding the depletion phase:
						-Acute fall of AMH as soon as 15 days after the start of CT.
						-Undetectable values of AMH at the end of CT in both protocols.
						Different recovery phases according to protocol:
						-Very low or undetectable AMH levels in the alkylating group 1 year post-CT.
						-Return to pre-treatment values of AMH as soon as the 6th month of follow-up in the non-alkylating group.
**Rosendahl, 2010 **[53]	Lymphoma, Breast cancer, Ewing sarcoma	7 different protocols	30 (19–35)	Before CT, one week after each CT or every 2 weeks until 16 weeks of treatment, once a month until one year after first CT	17 (13 alkylating protocol, 4 non alkylating protocol)	-Acute fall of AMH levels as soon as one week after the first cycle of CT.
						-Undetectable values of AMH in most patients at the end of CT.
						-Significantly lower AMH one year after the end of CT in case of alkytlating protocols than in case of non alkylating ones.
**Yu, 2010 **[29]	Breast cancer	3 different protocols all including alkylating agents	37 (27–40)	Before CT, 6, 12, 36 and 52 weeks after first CT	26	-Significant fall of AMH levels 6 weeks after the start of CT.
						-Undetectable values of AMH in most patients at the end of CT.
						-Undetectable AMH levels in all patient but one 52 weeks after first CT.
**Anderson, 2011 **[25]	Early stage breast cancer	7 protocols all including alkylating agents	41 (28,6-52,7)	Before CT, 2,3,4 and 5 years post CT	42	-Lower serum AMH levels, 2–5 years after CT, than pretreatment ones.
						-Undetectable AMH in most women, 2–5 years post-CT.
						-Pretreatment AMH level is strongly predictive of long term ovarian function after CT.
**Dillon, 2013 **[52]	Different types of cancer	Different protocols	26,1 (15 – 35,9)	Before CT, Every 3 month during and after treatment	46 (33 alkylating protocol)	Difference between protocols regarding the depletion phase:
						-Significant fall of AMH levels 3 months after initiation of CT in alkylating and non-alkylating groups
						-Lower AMH at the end of treatment with alkylating agents compared with unexposed participants
						Different recovery phases according to protocol and to pretreatment AMH level:
						-Rate of recovery of AMH 9 months after CT higher in non alkylating protocol than in alkylating protocol
						-Rate of recovery of AMH 9 months after CT higher when pre-treatment AMH was >2 ng/ml than when it was ≤ 2 ng/m
**Anderson, 2013 **[26]	Early stage breast cancer	8 protocols, 7 including alkylating agents	42,6 (23,3–52,5)	Before CT, after 1 or 2 cycles of CT, 1 and 2 years post CT	59	-Significant fall of AMH after 1 cycle of CT.
						-Undetectable values of AMH in most patients after 2 or more cycles of CT and at 1 year post CT.
						-Pretreatment AMH level is strongly predictive of post CT ovarian function (menses) at 1 and 2 years post CT.
**Henry, 2013 **[51]	Breast cancer	5 protocols all including alkylating agents except for 1 patient	41 (25–50)	Before CT, 1 month post CT, 1 year post CT.	27	-Undetectable values of AMH in all patients 1 month post CT.
						-Undetectable AMH levels in most women 1 year post CT.

**Table 3:** Summary of studies with longitudinal follow-up of serum AMH levels in young girls undergoing chemotherapy (CT) for childhood cancer

**Study**	**Cancer**	**Treatment**	**Age (range)**	**Times of AMH measurement**	**Number of patients**	**Conclusions about AMH follow up**
**Broughman, 2012 **[54]	Different type of cancer	Different protocols (low/ medium/high gonadotoxic protocols)	4,4 (0,3-15)	Before CT, after each CT course, between 2 and 12 months post CT, >12 months post CT	22 (9 high gonadotoxic protocol, 13 medium or low gonadotoxic protocol)	No difference between protocols regarding the depletion phase:
						-Significant fall of AMH levels from the third cycle of CT in all protocols.
						-Undetectable values of AMH in most patients at the end of CT.
						Different recovery phases according to protocol:
						-Very low or undetectable AMH levels in the high gonadotoxic group more than 12 months post CT.
						-Return to pre-treatment AMH values as soon as the 6th month of follow-up in the medium/low group risk.
**Mörse, 2013 **[55]	Different type of cancer	Different protocols	9,5 (4,5-16,5)	Before CT, Every 3 month during and after CT	34 (27 alkylating protocol)	No difference between protocols regarding the depletion phase:
						-Significant fall of AMH levels 3 months after initiation of CT.
						Different recovery phases according to protocol:
						-Very low or undetectable AMH levels in the group with irradiation below the diaphragm and/or stem cell transplantation 18 month post follow-up.
						-Return to pre-treatment or higher AMH values as soon as the 15th month of follow-up in acute lymphatic leukemia group.

A marked and prompt fall in serum AMH levels as early as the first month of chemotherapy has been described in four longitudinal studies [24,26,53,54]. Decanter et al. [24] investigated 30 young lymphoma patients (mean age: 24 years) divided into two groups according to the presence or not of alkylating agents in their chemotherapy protocol. AMH levels decreased significantly 15 days after the start of treatment, even in the non-alkylating group. Rosendahl et al. [53] found the same results as soon as week 1 after initiation of chemotherapy in 17 patients treated for lymphoma, breast cancer or Ewing sarcoma, of which 13 had an ovary removed for cryopreservation. Brougham et al. [54] recently described the same drastic AMH fall in 22 prepubertal and pubertal girls (median age: 4,4 years) treated by various chemotherapy or radiotherapy protocols for different types of cancer as did Anderson in 59 early breast cancer patient (mean age: 42.6) as soon as one cycle of chemotherapy [26]. Mörse et al. [55] and Dillon et al. [52] described this fall of AMH but only three months after the first cycle of chemotherapy in, respectively, 34 young patients (mean age: 9,5 years) and 46 patients (mean age: 26,1 years) treated for different kinds of cancer. At the end of chemotherapy, these studies showed significantly decreased or undetectable AMH levels, independently of protocols. The study of Dillon et al. [52] is the only study to prove an association between alkylating agent exposure and post-therapy impairment of AMH but in a very heterogeneous group.

The same significantly decreased or undetectable AMH levels at the end of chemotherapy was obtained in studies conducted in breast cancer patients by Anderson et al., Anders et al., Yu et al. and Henry et al. [27-29,51]. Anderson et al. in 2006 [28] investigated 50 breast cancer patients (mean age: 41 years) who were treated by different regimens of adjuvant chemotherapy, all including cyclophosphamide. In most of the patients, AMH levels remained undetectable after treatment. Anders et al., Yu et al. and Henry et al. confirmed these results in 44, 26 and 27 premenopausal breast cancer patients, respectively [27,29]. The drastic AMH fall just after the beginning of chemotherapy suggests that most of the growing follicles are damaged. These results are in keeping with those previously described in the animal models with alkylating agents [14-16].

Conversely, the pattern of the ovarian recovery phase greatly differs depending on the protocols. Decanter et al. [24] showed different ovarian recovery patterns between the groups with or without alkylating agents. Patients who were treated with a non-alkylating regimen recovered their pre-treatment AMH levels, as early as the 6th month of follow-up. In contrast, in the alkylating regimen group, AMH values during all the follow-up remained significantly different from the pre-treatment value, with very low or undetectable levels. Rosendahl et al. [53] also reported significantly higher AMH values in patients who did not receive alkylating agents. All women treated for breast cancer received alkylating agents and, in the three studies, serum AMH levels after chemotherapy remained very low or undetectable in all of them. Likewise, in a study by Brougham et al. [54], different recovery phases were also described between the high gonadotoxic risk group and the medium/low risk group. These groups were defined depending on chemotherapy drugs, cumulative dose and exposure (or not) to radiotherapy involving the ovaries. Indeed, AMH remained undetectable even 3 years after the end of chemotherapy in the high risk group. In contrast, in the low/medium risk group, the recovery occurred as soon as 6 months after treatment. The work of Mörse et al. [55] confirmed these results with undetectable AMH levels 18 months after the beginning of chemotherapy in patients who received irradiation below the diaphragm and/or stem cell transplantation. But after 15 months of follow-up in the 7 patients treated for acute lymphatic leukaemia (5 with alkylating agents), AMH levels returned to pre-treatment or higher values. In this last study, distinction between high and low gonadotoxic risk was not possible because of the heterogeneity of the recruitment and the small number of patients in each group. Finally, a recent work of Dillon et al. [52] concluded that the rate of recovery of AMH after the end of chemotherapy was impacted by the alkylator use. Almost all women treated for breast cancer received alkylating agents and, in the five studies, serum AMH levels remained very low or undetectable during the entire follow-up after chemotherapy [26-29,51].

Only the dynamics of AMH level during the recovery phase can discriminate between protocols and suggest different degrees and/or mechanisms of ovariotoxicity. Presumably, the primordial follicle pool is less damaged in the case of non-alkylating protocols, therefore allowing the rapid emergence of newly growing and AMH-secreting follicles. Intriguingly, AMH levels decreased drastically just after the beginning of chemotherapy even in the non-alkylating protocol group. Whether this fall of AMH could reflect either the follicle depletion or a functional granulosa cell impairment remains to be established.

### Clinical relevance of AMH follow-up in young women exposed to chemotherapy

Infertility and early menopause are associated with chemotherapy even in women who resumed menses after treatment [56]. The probability of early menopause and infertility depends on the type of cancer, the treatment, and the age at diagnosis [56]. Relationships between post-chemotherapy AMH levels and ovarian function, as indicated by menstrual activity, are difficult to establish. Indeed, among patients in whom AMH levels remained undetectable at least one year after the end of chemotherapy, a significant number had already recovered spontaneous menses [24,25,27-29,53,57]. Conversely, all the patients with persisting amenorrhea at one year of follow-up had undetectable AMH levels [24,25,28,57]. It may be that ELISA AMH assays currently available are not sensitive enough to detect very low values of AMH in women who recovered menses despite “undetectable” AMH level in the present dosage. There are very limited data about AMH and spontaneous conception. One study, by Hagen et al., showed no reduced fecundity in young women with a low AMH level compared to normal AMH [58] but another study showed that rather older women did show a relationship between AMH and spontaneous fertility [59]. In addition, it is now well established that a low AMH level (<0.7 μg/l) is of poor prognosis in assisted reproductive therapy [32]. Nevertheless, whether a low post-chemotherapy AMH level predicts the same low chances of conception remains to be elucidated.

Anderson et al. recently showed that pre-treatment AMH level is the strongest predictor (better than age) of menstrual activity at 1, 2 and 4–5 years of follow-up in two studies including 59 and 42 breast cancer patients [26,28]. Similarly, Rosendahl et al. [53] showed that high pre-treatment AMH levels were predictive of higher AMH levels during recovery of ovarian function after chemotherapy, independently of patient’s age, removal or not of one ovary and type of chemotherapy. In the same way, Dillon et al. [52], very recently, showed a better rate of AMH recovery after the end of chemotherapy when pre-treatment AMH level was over 2 ng/ml. Likewise, Anders et al. [27] reported that pre-treatment AMH concentration was lower in women treated for breast cancer who were amenorrheic one year after chemotherapy, although Yu et al. have reported no such difference [29]. Noteworthy, pre-treatment AMH level can be lower in haematological malignancies before chemotherapy [48]. The possible causes of the reduced AMH level in haematological malignancies prior to chemotherapy are still unclear. Maybe a functional impairment of granulosa cells by compromised general health or systemic inflammation can explain or contribute to the reduced AMH level. Very recently, Dorp et al. [60] described specifically these reduced AMH levels in very young girls with newly diagnosed cancer. They hypothesized that a decrease in AMH production, an increased metabolism of AMH or a more rapid decline of the primordial follicle pool might be involved in this reduced AMH level. This must be taken into account for fertility preservation counselling.

Thus, the pre-treatment AMH level may predict the long-term ovarian function after chemotherapy but the prognosis of conception when the AMH level is low after chemotherapy needs to be ascertained. Indeed, even in young healthy women, the low concentration of serum AMH is not predictive of reduced fecundability [58]. Only a long-term and systematic follow-up of ovarian reserve by AMH and of spontaneous fertility in a large population will allow these issues to be addressed.

## Conclusions

Serum AMH is a very convenient and sensitive indicator of follicular depletion and recovery in young women during and after chemotherapy. Furthermore, it allows for the detection of differences in ovarian toxicity between chemotherapy regimens. A systematic follow-up of AMH levels in women undergoing gonadotoxic treatment enables the evaluation of the degree of follicular depletion and recovery. It may help to better understand the ovarian toxicity mechanisms of chemotherapy. Furthermore, it could be useful to elaborate fertility preservation strategies, fertility counseling and future family planning, but we must be careful for now since there is limited data on the prediction of serum AMH on ongoing pregnancy in cancer survivors.

## Abbreviations

AFC: Antral Follicle Count; AMH: Anti Müllerian Hormone; DSL: Diagnostic Systems Laboratories; FSH: Follicle Stimulating Hormone; MIS: Müllerian Inhibiting Substance.

## Competing interests

The authors declare that they have no competing interests.

## Authors’ contribution

MP contributed to acquisition of data, analysis and interpretation of data and has been involved in drafting the manuscript. CD has been involved in revising the manuscript critically for important intellectual content and has given final approval of the version to be published. Both authors read and approved the final manuscript.

## Authors’ information

CD is responsible of the feminine preservation center in the north of France in Lille. She developed in her center a systematic follow up of ovarian reserve of women undergoing chemotherapy. MP works in the same feminine fertility preservation center and is also involved in women’s follow up.
